# Use of the subcutaneous ureteral bypass system (SIDUS) in dogs and cats: a case series study

**DOI:** 10.29374/2527-2179.bjvm005926

**Published:** 2026-09-22

**Authors:** Letícia Alves Wrobel, Fabiana Antunes Andrade, Eloísa Muehlbauer, Mariana Etchepare, Renato Nisihara

**Affiliations:** 1 Programa de Pós-Graduação em Biotecnologia Industrial, Universidade Positivo, Curitiba, PR, Brazil; 2 Departamento de Medicina Veterinária, Universidade Positivo, Curitiba, PR, Brazil

**Keywords:** azotemia, dogs, cats, ureteral obstruction, subcutaneous ureteral bypass, azotemia, cães, gatos, obstrução ureteral, bypass ureteral subcutâneo

## Abstract

Subcutaneous ureteral bypass (SIDUS) has become an established surgical alternative for the management of ureteral obstruction in cats and dogs, providing urinary diversion and facilitating renal decompression. This retrospective cohort study evaluated the clinical, biochemical, and microbiological outcomes of 17 animals that underwent SIDUS implantation and were followed for up to 90 days. Data were retrospectively obtained from medical records, including clinical assessments, laboratory tests, and urine cultures. Serum creatinine concentrations decreased significantly from a preoperative median of 10.0 to 6.0 mg/dL at 24 h after surgery (*p* < 0.001) and remained at a median of 1.8 mg/dL at 60 and 90 days. Urea and phosphorus concentrations showed similar reductions following implantation. Leukocyte counts exhibited mild postoperative variation, consistent with a possible inflammatory response. Urine cultures were predominantly negative throughout follow-up, and positive cultures were sporadic and not accompanied by reported clinical signs or deterioration of laboratory parameters. Despite the small sample size and limited follow-up period, these findings indicate substantial improvement in renal biochemical parameters following SIDUS implantation, with few clinically relevant urinary tract infections observed during the study period.

## Resumo

O bypass ureteral subcutâneo (SIDUS) tornou-se uma alternativa cirúrgica estabelecida para o manejo da obstrução ureteral em gatos e cães, proporcionando o desvio do fluxo urinário e facilitando a descompressão renal. Este estudo de coorte retrospectivo avaliou os desfechos clínicos, bioquímicos e microbiológicos de 17 animais submetidos à implantação do SIDUS e acompanhados por até 90 dias. Os dados foram obtidos retrospectivamente a partir dos prontuários médicos, incluindo avaliações clínicas, exames laboratoriais e culturas de urina. As concentrações séricas de creatinina diminuíram significativamente de uma mediana pré-operatória de 10,0 para 6,0 mg/dL 24 horas após a cirurgia (*p* < 0,001), permanecendo em uma mediana de 1,8 mg/dL aos 60 e 90 dias. As concentrações de ureia e fósforo apresentaram reduções semelhantes após a implantação. As contagens de leucócitos apresentaram discreta variação no período pós-operatório, compatível com uma possível resposta inflamatória. As culturas de urina foram predominantemente negativas durante todo o período de acompanhamento, e as positivas foram esporádicas e não estiveram acompanhadas de sinais clínicos relatados nem de deterioração dos parâmetros laboratoriais. Apesar do pequeno tamanho amostral e do período limitado de acompanhamento, esses achados indicam melhora substancial dos parâmetros bioquímicos relacionados à função renal após a implantação do SIDUS, com poucas infecções do trato urinário clinicamente relevantes observadas durante o período do estudo**.**

## Introduction

Ureteral obstruction in cats and dogs is an increasingly recognized challenge in small animal practice and may lead to hydronephrosis, pyelonephritis, progressive loss of renal function, and, ultimately, chronic kidney disease. Common underlying causes include urolithiasis, ureteral strictures, inflammatory conditions, and neoplasia. Severe obstruction can result in azotemia and obstructive acute kidney injury. Therefore, early diagnosis and timely intervention are critical for relieving obstruction, preserving renal function, and improving clinical outcomes.

Over the past several decades, the management of ureteral obstruction has evolved from predominantly conservative or temporary measures, which may be associated with substantial complication rates, toward minimally invasive interventional techniques. Among these, ureteral stenting and the subcutaneous ureteral bypass system (SIDUS) have become important treatment options for cats and dogs with ureteral obstruction. SIDUS has been associated with high clinical success rates and improvement or stabilization of renal function, particularly in patients with refractory or multiple ureteral obstructions (Ahmed, 2021; Berent & Weisse, 2011; Deprey, 2021).

Despite its clinical benefits, implantation of biomaterials within the urinary tract may facilitate bacterial adhesion, colonization, and biofilm formation. However, recent evidence suggests that positive urine culture results do not necessarily indicate active infection, as subclinical bacterial colonization is common. Therefore, clinical findings should be interpreted in conjunction with laboratory and microbiological results (Deprey, 2021; Epstein, 2021; Weese et al., 2019).

In this context, the present study aimed to evaluate the clinical, laboratory, and microbiological outcomes of cats and dogs undergoing SIDUS implantation during a follow-up period of up to 90 days. Specifically, the study sought to characterize changes in renal function and assess the occurrence of urinary tract infection following implantation.

## Materials and methods

### Ethical aspects

This study was approved by the Institutional Animal Care and Use Committee of Universidade Positivo (CEUA/UP), under protocol number 32/2024.

### Study design and patient selection

A retrospective cohort study was conducted based on a review of the medical records of cats and dogs treated at a private veterinary hospital in Curitiba, Brazil, between January 2021 and June 2024. Animals that underwent SIDUS implantation and were monitored by the nephrology service for 90 days were eligible for inclusion.

Inclusion required the availability of urinalysis and urine culture with antimicrobial susceptibility testing during the preoperative period and at 30, 60, and 90 days postoperatively. Complete blood count, serum creatinine, urea, phosphorus, venous blood gas analysis with electrolyte measurements, and abdominal ultrasonography were also required at these time points. Animals that underwent emergency surgery without a preoperative urine culture, had laboratory or imaging examinations performed outside the hospital, were not followed by the same nephrology team, or did not adhere to the established device-maintenance protocol were excluded.

### Data collection

Data were retrospectively extracted from electronic medical records and included demographic characteristics (species, age, sex, reproductive status, and body weight), laboratory test results, and imaging findings. Urine culture and corresponding antimicrobial susceptibility testing results were recorded for the preoperative period, immediate postoperative period, and subsequent follow-up evaluations. Abdominal ultrasonography reports were reviewed for findings associated with ureteral obstruction, including renal pelvic dilation, ureteral wall thickening, ureteroliths, and evidence of extrinsic compression.

### Surgical technique and postoperative management

Before surgery, patients were fasted for 8-12 h and received intravenous fluid therapy for maintenance and correction of fluid and electrolyte abnormalities as clinically indicated. Anesthesia was induced with propofol and fentanyl and maintained with isoflurane in oxygen. Intraoperative monitoring included pulse oximetry, capnography, electrocardiography, and invasive blood pressure measurement.

The surgical procedure consisted of a ventral midline celiotomy, placement of ureteral catheters under fluoroscopic guidance, renal fixation, cystostomy for placement of the bladder catheter, and connection of the components using an “X” connector. The subcutaneous port was secured to the abdominal fascia, and system patency was assessed by infusion of sterile saline under fluoroscopic guidance. The abdomen was subsequently closed in anatomical layers.

Postoperatively, patients underwent intensive monitoring and received multimodal analgesia and antimicrobial therapy when clinically indicated, with antimicrobial selection guided by urine culture results. SIDUS was flushed with heparinized saline at 24 h, 7 days, and 30 days after surgery and subsequently at 3-month intervals. Follow-up evaluations included laboratory testing, abdominal ultrasonography, urinalysis, and urine culture.

### Statistical analysis

Data were entered into electronic spreadsheets and analyzed using GraphPad Prism. The normality of continuous variables was assessed using the Shapiro-Wilk test. Continuous variables were summarized as medians and interquartile ranges (IQRs). Comparisons between two related time points were performed using the Wilcoxon signed-rank test. A two-sided *p* < 0.05 was considered statistically significant.

## Results

Seventeen animals that underwent SIDUS implantation were included in the study, comprising 10 dogs (58.8%) and 7 cats (41.2%). The study population included eight females (47.1%) and nine males (52.9%). Most animals were younger than 10 years (15/17; 88.2%), and 10 animals (58.8%) were neutered.

Serum creatinine concentrations were markedly increased preoperatively, with a median of 10.0 mg/dL. At 24 h after SIDUS implantation, the median concentration decreased significantly (*p* < 0.001) to 6.0 mg/dL. A further reduction was observed at 30 days, when the median concentration reached 2.0 mg/dL. Median creatinine concentrations remained stable at 1.8 mg/dL at both 60 and 90 days, indicating sustained improvement in renal function following ureteral decompression (Figure 1).

**Figure 1:**
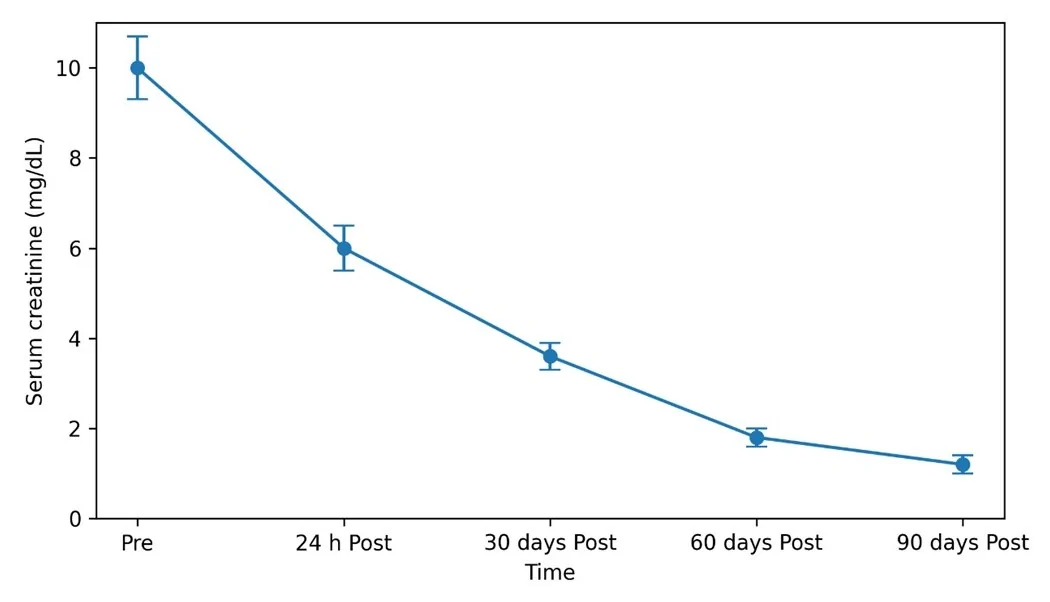
Evolution of serum creatinine concentrations over the 90-day follow-up period.

*Note*. Data points represent median values, and error bars indicate the IQR. Results are presented collectively for cats and dogs that underwent SIDUS implantation and were followed for 90 days.

Urea and phosphorus concentrations showed a similar pattern, with significant reductions after SIDUS implantation. Leukocyte counts exhibited mild postoperative variation, but no statistically significant differences were detected (*p* > 0.05). No left shift was observed during follow-up. Therefore, the observed leukocyte changes were interpreted as an inflammatory response associated with surgical manipulation and implantation of the device.

Urine cultures were predominantly negative throughout the follow-up period. Positive cultures occurred sporadically and were not accompanied by reported clinical signs or deterioration of laboratory parameters.

## Discussion

The present study demonstrated rapid improvement in renal biochemical parameters following SIDUS implantation, with serum creatinine concentrations decreasing significantly within 24 h after surgery and remaining substantially lower throughout the 60- and 90-day follow-up periods. Similar trends were observed for urea and phosphorus concentrations, supporting the beneficial effect of ureteral decompression on biochemical markers of renal function. These findings are consistent with previous reports (Ahmed, 2021; Butty, 2023; Deprey, 2021).

The metabolic abnormalities identified before surgery were consistent with the severity of obstructive acute kidney injury in this population. Hypokalemia was the predominant potassium abnormality, occurring in 76% of animals, whereas hyperkalemia was identified in 24%. The predominance of hypokalemia may reflect several factors, including prior fluid administration and alterations associated with the post-obstructive phase, as described in recent studies (Hecht, 2024).

The microbiological findings provide additional insight into urinary tract complications following SIDUS implantation. Most urine cultures remained negative during follow-up, suggesting that SIDUS implantation, when combined with appropriate device maintenance and monitoring, was not necessarily associated with an increased incidence of clinically relevant urinary tract infection. These findings highlight the importance of interpreting urine culture results in conjunction with clinical findings and laboratory abnormalities, in accordance with ISCAID guidelines (Epstein, 2021; Weese et al., 2019).

The study limitations include the small sample size and limited 90-day follow-up, which preclude conclusions regarding late-onset complications such as device mineralization and mechanical failure. Multicenter studies involving larger populations and longer follow-up periods are warranted to address these limitations and better characterize long-term outcomes.

## Conclusions

SIDUS was an effective treatment for ureteral obstruction in cats and dogs, resulting in statistically significant and sustained improvements in biochemical markers of renal function during the first 90 days after implantation. These findings support the clinical use of SIDUS when combined with appropriate follow-up and device maintenance.
